# Current concepts in the pathophysiology of glaucoma

**DOI:** 10.4103/0301-4738.53049

**Published:** 2009

**Authors:** Renu Agarwal, Suresh K Gupta, Puneet Agarwal, Rohit Saxena, Shyam S Agrawal

**Affiliations:** Department of Ocular Pharmacology, Delhi Institute of Pharmaceutical Sciences and Research, New Delhi, India; 1 Dr. Rajendra Prasad Center for Ophthalmic Sciences, New Delhi, India

**Keywords:** Glaucoma, pathophysiology, retinal ganglion cells

## Abstract

Glaucoma, the second leading cause of blindness, is characterized by changes in the optic disc and visual field defects. The elevated intraocular pressure was considered the prime factor responsible for the glaucomatous optic neuropathy involving death of retinal ganglion cells and their axons. Extensive investigations into the pathophysiology of glaucoma now reveal the role of multiple factors in the development of retinal ganglion cell death. A better understanding of the pathophysiological mechanisms involved in the onset and progression of glaucomatous optic neuropathy is crucial in the development of better therapeutic options. This review is an effort to summarize the current concepts in the pathophysiology of glaucoma so that newer therapeutic targets can be recognized.

The literature available in the National Medical Library and online Pubmed search engine was used for literature review.

Glaucoma, a leading cause of irreversible visual loss, is characterized by loss of retinal ganglion cells (RGC) and their axons over a period of many years. Glaucomatous optic neuropathy is characterized by changes in the optic disc and visual field defects.[1,2] The morphologic changes in the optic disc are in the form of thinning of neuroretinal rim, pallor and progressive cupping of the optic disc. The hemorrhage-associated retinal nerve fiber layer defects precede measurable changes of the optic disc configuration.[3] The visual field defects in glaucoma are often detected only after 40% of the axons are lost.[4]

The pathophysiology of glaucomatous optic neuropathy is not well understood. Whether the site of primary damage is the ganglion cell body or their axons remains fiercely debatable. Irrespective of the initial site of neuronal injury and mechanisms involved, the terminal outcome is the death of RGCs and their axons leading to irreversible visual loss. This paper presents a review of the various mechanisms involved in the development of glaucomatous optic neuropathy. The literature published during 1984 to 2006 was reviewed except for the two research papers (Lucas and Newhouse, 1957 and Onley, 1969) which were included to show that the important role of glutamate toxicity in glaucomatous optic atrophy has been suspected for a long time. All the literature reviewed was obtained from the National Medical Library and online Pubmed search engine. All articles referenced were published in English language journals.

## Multifactorial pathogenesis of glaucoma

Glaucoma is a heterogeneous group of diseases and the pathophysiology of glaucoma is believed to be multifactorial. Multiple factors acting either on cell bodies or their axons are believed to lead to RGC death. According to various theories put forth, factors like elevated intraocular pressure (IOP) and vascular dysregulation primarily contribute to the initial insult during glaucomatous atrophy in the form of obstruction to axoplasmic flow within the RGC axons at the lamina cribrosa, altered optic nerve microcirculation at the level of lamina and changes in the laminar glial and connective tissue. The factors leading to secondary insult include excitotoxic damage caused by glutamate or glycine released from injured neurons and oxidative damage caused by over-production of nitric oxide (NO) and other reactive oxygen species. Whatever may be the primary and secondary factors, the end result in glaucomatous eyes is the dysfunction and death of RGCs leading to irreversible visual loss, as a result of a complex interplay of multiple factors rather than any one of them functioning individually.[5]

### Neuronal loss in glaucoma by apoptosis

The characteristic change in the optic nerve head in glaucoma is a “cupping” of the optic disc where ganglion cell axons have been lost. The death of the axons is associated with a loss of ganglion cell bodies in the retina and ganglion cell axon terminals in the dorsal lateral geniculate body. Death of RGCs in glaucomatous human eyes and experimental animal models of glaucoma takes place by apoptosis,[6,7] which is also the means of eliminating 50% of the RGCs during normal developmental organization of the visual pathway.[8] Apoptosis is a process of programmed cell death in the absence of inflammation, characterized by DNA fragmentation, chromosome clumping, cell shrinkage and membrane blebbing.[9] Nuclear damage is followed by breaking down of the cell into multiple membrane-bound vesicles which are engulfed by neighboring cells. Some researchers have suggested preferential loss of larger ganglion cells in the retina belonging to parasol and midget cell classes[4,10–12] but this issue still remains debatable.[13] Although there are compelling evidences showing apoptosis as the primary and early mechanism of ganglion cell death in glaucoma, necrosis is also a contributory mechanism in the late phase, evidence to which was observed in rats subjected to optic nerve transection.[14]

The caspases, a family of cysteine aspartyl-specific proteases have emerged as the central regulators of apoptosis. These enzymes are present as inactive zymogens and once activated initiate an ordered cascade leading to proteolysis of key cytosolic and nuclear components and eventual destruction of the cell.[15] The activation of caspases involves an extrinsic and an intrinsic pathway. The extrinsic pathway involves interaction of specific ligands such as tumor necrosis factor-alpha (TNF-A) with the proapoptotic cell surface receptors while the intrinsic pathway is regulated by proapoptotic molecules released from the mitichondrion.[16] In rats subjected to axotomy, intraocular application of various caspase inhibitors rescued up to 34% of RGCs that would otherwise have died 14 days after optic nerve transection. Involvement of caspases in RGC apoptosis suggests the possible role of additional interventional strategies using caspase inhibitors.[17]

While the evidences suggest the central role of caspases in adult RGC death, there is a growing body of evidence suggesting involvement of caspase-independent pathways in RGC death under certain conditions. Spalding *et al*. have reported significant RGC death within 24 h of superior colliculus ablation in neonatal rats which was not inhibited by general or specific caspase inhibitors.[18]

### Elevated intraocular pressure - the prime factor?

Until recently it was believed that elevated IOP plays a major role in RGC apoptosis and it is also true that reduction of elevated IOP often helps in slowing down the progression of degenerative changes in glaucoma. However, among glaucoma patients only one-third to half of all glaucoma patients have elevated IOP at the initial stages.[1,19,20] On an average, 30-40% of patients with glaucomatous visual field defects are being diagnosed as having normal tension glaucoma (NTG) in peripheral clinics.[21] Therefore, elevated IOP is now believed to be an important but not the only factor responsible for optic nerve damage.

Elevated IOP often results from alterations in aqueous humor dynamics due to changes in trabecular meshwork leading to impaired drainage of aqueous. The trabecular meshwork has been shown to exhibit cytoskeletal changes in cells,[22] altered cellularity[23,24] and changes in extracellular matrix (ECM).[25–27] Several investigators have studied the association of RGC loss and elevated IOP. A significant positive correlation has been observed between change in IOP and RGC death in glaucomatous rats.[28–30] A positive correlation has also been observed between the level and duration of elevated IOP and RGC axon loss.[29,30] Loss of half of the ganglion cells takes place during the initial two to three months of IOP elevation.[31–33] RGC death in experimental glaucoma has been shown to occur by the process of apoptosis[7,34] and IOP elevation can directly induce RGC death by apoptosis.[32] Results of a number of experiments suggest that RGC death after exposure to elevated IOP takes place in two phases. The first phase lasts for about three weeks, with loss of approximately 12% RGCs per week. This is believed to be followed by a second slower phase of neuronal loss.[32] The primary mechanism of neuronal loss in the initial phase is apoptosis[35] while in the second phase neuronal loss is due to toxic effects of the primary degenerating neurons in addition to continuing exposure to elevated IOP.[30]

### Molecular mechanisms of retinal ganglion cell apoptosis in response to elevated intraocular pressure

Cellular responses to changes in IOP, leading to apoptosis of RGCs are not well understood. A possible mechanism of RGC apoptosis seems to be related to changes in extracelluar matrix components in the retina of glaucomatous eyes in response to elevated IOP. Extensive remodeling of the ECM, including collagen I and IV, transforming growth factor-ß2 (TGF-ß2), and matrix metalloproteinase (MMP)-1 have been detected in glaucomatous eyes.[36–38] ECM is responsible for providing adherence signals thereby controlling the cell functions and cell survival.[39] Therefore, changes in specific ECM components can interrupt cell-cell and cell-ECM interactions, leading to cell death by apoptosis.

MMPs are the major matrix-degrading enzymes. In a recent study enhanced MMP-9 activity was detected in apoptotic RGCs along with decreased deposition of laminin in the RGC layer suggesting increased degradation of the ECM at the retinal site in response to exposure to elevated IOP.[40] Laminin is an important ECM component, which facilitates cell adherence and survival by interacting with cellular integrins. Disintegration and loss of laminin as a result of increased amount of proteases such as MMP-9 leads to deficient cell-ECM communication thereby favoring cell loss by apoptosis.[41] Thus the results of the above-mentioned studies indicate that upon exposure to elevated IOP there is increased secretion of MMP-9 from RGCs leading to increased degradation of laminin and apoptosis. According to another explanation elevated IOP causes mechanical damage to RGC axons in the region of optic nerve head which progresses to retrograde damage of the RGC body. Damage to RGC bodies leads to enhanced secretion of MMPs, which in turn causes ECM changes and apoptosis. Another alternative theory suggests that increased MMP expression as a result of exposure to elevated IOP could be mediated indirectly by the excitatory neurotransmitter glutamate. Upregulation of glutamate receptors in retinal cells was found to be associated with increased MMP-9 expression.[42] It has also been demonstrated that exposure to elevated IOP leads to activation of retinal astrocytes.[43] These activated astrocytes release MMPs to bring about changes in the pattern of matrix remodeling.[44]

Growth factors and their receptors are known to regulate cellular functions, cytoskeletal organizations and components of ECM in ocular tissue. Trabecular meshwork, optic nerve astrocytes as well as lamina cribrosa cells express a wide variety of growth factors such as neurotrophin factor and TGF-ß2. These growth factors may play an important role by affecting the normal development and cellular functions in the trabecular meshwork as well as retina. In the retina retrograde axoplasmic transport block, as a result of elevated IOP can deprive the RGCs of the supply of brain-derived neurotrophin factor (BDNF), important for regulating cell metabolism and cell survival. Deficiency of BDNF can further lead to progression of RGC apoptosis.[45] These effects seem to be further modulated by increased release of TGF-ß2 by activated astrocytes in response to elevated IOP.[37]

**Figure 1:**
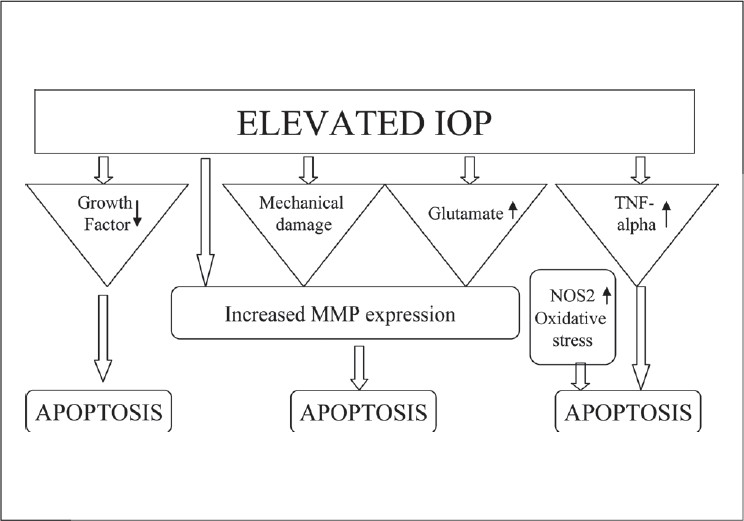
Mechanisms involved in glaucomatous RGC apoptosis secondary to elevated IOP. TNF-A - Tumor necrosis factor-alpha, MMP - Matrix metalloproteinase, NOS-2 - Nitric oxide synthase-2

Nakazawa *et al.*, have now demonstrated rapid upregulation of TNF-A in rats with experimentally induced elevated IOP and this was followed sequentially by microglial activation, loss of optic nerve oligodendrocytes, and delayed loss of RGCs.[46] An upregulation of TNF-A in the astrocytes was also detected in human glaucomatous optic nerve head and this expression was found to parallel the progression of neurodegeneration. TNF-A stimulation seems to contribute to neuronal damage by both a direct effect on the axons of the RGCs and by inducing nitric oxide synthase (NOS)-2 in astrocytes.[47] A summary of mechanisms involved in RGC apoptosis secondary to elevated IOP is presented in Fig. 1.

### Vascular insufficiency: Another important factor

Clearly, elevated IOP plays a major role in RGC damage in glaucomatous eyes but therapeutic control of IOP in many patients is not sufficient to improve the visual functions and arrest the progression of the disease process.[48,49] Besides, glaucomatous changes have been observed in individuals with normal IOP. This suggests a critical role of other factors in the initiation and progression of glaucomatous changes.

A number of circumstantial evidences point towards an association between vascular insufficiency and glaucoma. A positive association of glaucoma has been observed with migraine[50,51] and peripheral vascular abnormalities[52,53] that involve dysregulation of cerebral and peripheral vasculature respectively. Increased sensitivity to endothelin-1-mediated vasoconstriction is implicated in these vascular abnormalities. The possible role of this vasoconstrictor is also suspected in the pathogenesis of glaucoma as increased levels of endothelin-1 have been detected in the aqueous humor and plasma of glaucoma patients.[54–57] Further evidences indicating a positive association between glaucoma and vascular insufficiency were provided by magnetic resonance imaging in glaucoma patients revealing pan-cerebral ischemia[58] and increased incidence of cerebral infarcts.[59] Aging is also considered an important risk factor for glaucoma and a progressive decline in cerebral and ocular perfusion has been observed with increasing age.[60,61] Based on these observations it can be hypothesized that neuronal damage in glaucoma represents a chronic anterior ischemic optic neuropathy.

In a healthy eye, a constant flow of blood is required in the retina and optic nerve head so as to meet the high metabolic needs in these vital parts of the eye. To maintain a constant rate of blood flow an efficient autoregulatory mechanism operates in arteries, arterioles and capillaries over a wide range of day-to-day fluctuations in ocular perfusion pressure that is dependent on both the systemic blood pressure and IOP.[62] These autoregulatory mechanisms are not as robust in aging individuals as in youth. Evidence of this can be observed in a study done by Matsuura and Kawai, showing robust choroidal hyperperfusion in response to experimentally induced ocular hypertension in young rats while in older rats a similar increase in choroidal perfusion was not observed.[63] Thus, deficient autoregulatory mechanisms leading to ischemia contribute to the development of glaucomatous neuronal damage with increasing age. Primary open angle glaucoma (POAG) and NTG patients have also shown a chronically reduced optic nerve head and retinal blood flow[64,65] especially in people with low systemic blood pressure leading to reduced ocular perfusion pressure.[66] Reduced diastolic perfusion pressure is now recognized as an important risk factor for POAG.[67]

**Figure 2:**
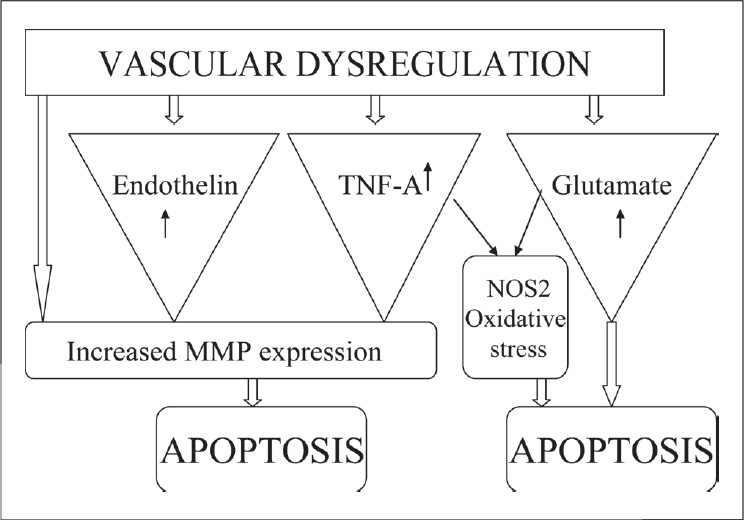
Mechanisms involved in glaucomatous RGC apoptosis secondary to vascular dysregulation. TNF-A - Tumor necrosis factor-alpha, MMP - Matrix metalloproteinase, NOS-2 - Nitric oxide synthase-2

The molecular mechanisms leading to RGC death due to vascular dysregulation are not clearly understood. The vascular insufficiency can directly damage and cause RGC apoptosis. Upregulation of MMP-9 expression in circulating leucocytes has been observed in patients with vasospastic NTG.[68] The upregulation of MMP can be a direct response to ischemic injury or it can be a secondary response to increased levels of endothelin and TNF-A. The MMP produced by the circulating leukocytes of these patients might be involved in the partial barrier breakdown and RGC damage[69] [Fig. 2].

### Role of glutamate in retinal ganglion cell death

Apoptotic cell death of RGCs has also been attributed to glutamate-mediated toxicity and upon exposure to hypoxic conditions retinal cells are known to release glutamate.[70] The amino acid glutamate is an essential neurotransmitter in the central nervous system and retina. Concentrations of glutamate higher than the physiological concentration are toxic to neurons depending upon the duration and extent of increase in concentration. The toxicity following acute exposure to high levels of glutamate is well documented. This toxicity of glutamate was first described by Lucas and Newhouse in 1957 who observed severe destruction of RGCs after subcutaneous injection of glutamate in young mice.[71] A similar glutamate-induced retinal toxicity was also observed by Onley and as the lesions developed upon exposure to excess levels of excitatory neurotransmitter they were described as excitotoxic.[72] A minor but chronic elevation of glutamate was also found to be toxic to ganglion cells.[73]

**Figure 3:**
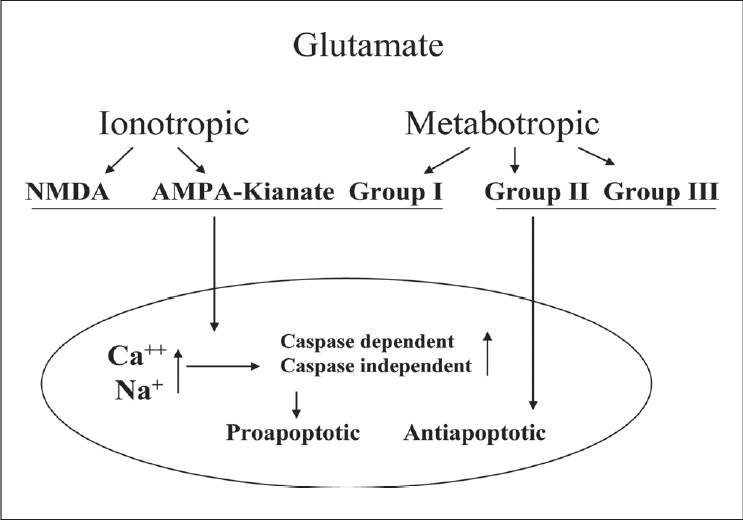
Glutamate excitotoxicity leading to RGC apoptosis

Glutamate-mediated neurotransmission is via ionotropic and metabotropic receptors [Fig. 3]. The ionotropic glutamate receptors include *N-*methyl-D-aspartate (NMDA), kianate (KA) and alpha-amino-3-hydroxy-5-methyl-4-isoxazolepropionic acid receptor (AMPA) receptors. The metabotropic receptors (mGluR) are G protein-linked receptors and are divided into three groups. Glutamate-induced excitotoxicity is primarily mediated by ionotropic NMDA subtype receptors.[74,75] NMDA receptor activation leads to opening of associated ion channels and the entry of extracellular Ca^++^ and Na^+^ into the neurons. Glutamate-mediated neuronal toxicity is dependent on the influx of extracellular Ca+, which in turn acts as second messenger to activate downstream signaling pathways finally leading to cell death.[74,76,77] Experimental administration of NMDA-antagonists has been shown to prevent glutamate-induced excitotoxicity.[78] In addition, tissue plasminogen activator (tPA) present in retinal neurons has also been suggested to be an important endogenous factor facilitating NMDA-mediated excitotoxicity. Although the mechanism of action of tPA is not well understood, it seems to be unrelated to conversion of plasminogen to plasmin.[79,80] Activation of metabotropic non-NMDA receptor has been demonstrated to protect against NMDA-mediated glutamate toxicity in primary culture of cerebellar neurons.[81]

Low concentrations of glutamate were found to activate Ca^++^-permeable AMPA-KA receptors in cultured RGCs, leading to increases in Ca^++^ and decreased RGC survival.[82] The KA receptors are non-synaptic and are uniquely positioned to report non-synaptic glutamate. At low concentrations of 1–5 μM kainate internal calcium concentration rises significantly without significant depolarization. This low concentration of kainate causes ganglion cell death, which could be inhibited by specific kainate receptor antagonists. The kainate-associated toxicity which results from excess influx of calcium can also be inhibited by polyamines and calcium phosphatase which suppress the calcium influx. Thus, activation of ionotropic glutamate receptors can produce neurotoxicity uncoupled from neuroexcitation.[83]

There is evidence that among the mGluR activation of group I mGluRs increases neuronal excitation, whereas that of group II and III reduces synaptic transmission. Therefore, group I mGluR antagonists and group II and III mGluR agonists are expected to provide neuroprotective effects.[84]

To maintain the physiological concentrations and to protect ganglion cells from excitotoxic cell death, appropriate removal of synaptic glutamate is required. Glial cells, especially the Muller cells and astrocytes, present around the synapses express the glutamate transporter which helps in clearing extracellular glutamate by transporting it to the interior of glial cells.[85] Within the glial cells the glutamate gets converted to glutamine in the presence of glutamine synthetase. Glutamine is non-toxic and is released by glial cells to be taken up by neuronal cells where it again gets converted to glutamate in the presence of glutaminase and thus the neurotransmitter stores are replenished and glutamate toxicity is prevented.

Deviations from this normal retinal glutamate/glutamine cycle have been observed in experimental models of glaucoma. In a glaucoma model of rat, Moreno *et al.*, reported significant decrease of retinal glutamate uptake, decreased activity of glutamine synthetase, significantly increased glutamine uptake and release and significantly increased glutaminase activity in oculohypertensive rats.[86] In one study immunohistochemical analysis of human glaucomatous and control eyes revealed decreased levels of both the glutamate transporter, excitatory amino acid transporter (EAAT)-1, and the NMDAR1 subtype suggesting that the loss of EAAT-1 in glaucoma may account for the elevated level of glutamate found in glaucomatous vitreous and lead to a compensatory downregulation of NMDAR-1. In the same study it was also observed that intravitreal injection of glial-derived neurotrophic factor (GDNF) in rats leads to elevated levels of both EAAT-1 and NMDAR-1.[87] A robust expression of a variant of glutamate transporter GLT-1c has been observed in RGC both in humans and rats. The GLT-1c in normal eyes is expressed only in photoreceptors. The induction of GLT-1c in RGCs in an attempt to protect themselves from toxic levels of glutamate indicates an important role of disturbed glutamate homeostasis in glaucomatous cell death.[88] Furthermore, alterations in the level of glutamate transporter m-RNA levels have been observed in both acute and chronic models of sublethal injuries in the eye and these alterations probably represent a transcriptionally regulated physiologic response.[89]

Some studies have suggested a correlation between elevated levels of glutamate in vitreous and apoptotic cell death in retina, however, others have reported no alterations in vitreous glutamate levels in glaucomatous eyes. Increased glutamate levels have been observed in mutant quail with a glaucoma-like disorder.[90] Elevated vitreous levels of glutamate were detected in an *in vivo* rat model of optic nerve ischemia.[91] In patients with retinal artery occlusion leading to acute retinal ischemia, the aqueous levels of glutamate were found to be elevated probably as a result of diffusion from the vitreous suggesting an important role of elevated levels of extracellular glutamate in ischemic retinal damage.[92] Dreyer *et al.*, reported significantly elevated vitreal glutamate levels in glaucomatous monkeys as compared to normal monkeys.[93] Later, in a study involving 26 monkeys Carter-Dawson *et al.*, reported that there were no significant differences in vitreal glutamate concentration between vitreous from normal control eyes and glaucomatous eyes, nor was there a significant difference in the results between the analyses performed in two independent laboratories.[94] Hare *et al.*, also reported no significant changes in vitreal glutamate levels in glaucomatous monkey eyes as compared to control eyes.[95] In another study involving eight glaucoma patients no significant changes were observed in vitreal glutamate levels by Honkanen.[96] These findings were in marked contrast to the report by Dreyer *et al*., and even after considering all possible factors that could have influenced the measurement of glutamate levels, the exceptionally high glutamate levels reported could not be explained. The validity of results of Dreyer *et al*., was therefore doubtful. A probe into the charges of scientific misconduct was initiated by Harvard University officials and the author quickly admitted fabricating some of his data. A heavy punishment of a 10-year debarment from receiving federal research funds was imposed.[97]

In spite of the controversial issues mentioned earlier glutamate excitotoxicity seems to play an important role in neurodegenerative changes in glaucoma. However, it remains to be determined whether the glutamate excitotoxicity is an initial response to elevated pressure and ischemia or whether the secondary response due to release from dying ganglion cells plays the more critical role.

### Role of nitric oxide in glaucoma

Nitric oxide plays an important and beneficial role in body function when secreted in physiological quantities, however, excess production of NO has been associated with a variety of non-neurological and neurological conditions including glaucoma. NOS produces NO by oxidation of L-arginine and has been detected in three isoforms.[98]

In normal human eyes the presence of NOS-1 has been detected in scattered astrocytes throughout the optic nerve head indicating that the NOS-1 is a constitutive enzyme in certain glia and NO serves functions as a physiological mediator between astrocytes or between astrocytes and axons. In patients with glaucoma a large number of cells show NOS-1 positivity on vitreal surface, in the remnant glial cells and in the cells in lamina cribrosa within glaucomatous tissue. Increased gene expression of the mRNA and presumably *de novo* synthesis of the NOS-1 isoform in astrocytes of the lamina cribrosa have also been observed. NOS-3 is also a constitutive enzyme present in the vascular endothelial cells in the prelaminar region of the optic nerve head in normal eyes and functions as a vasodilator. In glaucomatous eyes by causing vasodilation and increasing the blood flow NOS-3 induction can provide neuroprotective effects. The role of NOS-3 present in the astrocytes of glaucomatous optic nerve heads is not clearly known.[99]

NOS-2 is the inducible form of the enzyme (iNOS), which produces excessive quantities of NO under diverse conditions such as exposure to cytokines[100] and pressure.[101] Significant quantities of NOS-2 have been detected in the astrocytes[102] and microglia[103] at optic nerve head of glaucoma patients. Elevated NO levels have been observed in the aqueous humor of glaucoma patients[104] and a genetic association of iNOS and POAG has also been observed.[105] The animal experiments have also shown an association of elevated ocular NO levels with RGC death. Siu *et al.*, observed significantly elevated NO levels in the retina of rats, 35 days after laser treatment.[106] At this time point, post laser treatment, the rats had significantly elevated IOP and significantly reduced number of RGCs thus establishing an association between the excess production of NO and RGC death. Abnormalities of NO-containing cells in trabecular meshwork, Schlemm's canal and ciliary body have also been detected in patients with POAG, however, it is not known whether these abnormalities are the manifestations of glaucoma and its treatment or precede the development of disease.[107]

The molecular mechanisms of NOS-2 induction and production of neurotoxic quantities of NO have been studied in human optic nerve astrocyte culture. Besides elevated pressure various cytokines appear to play a key role in NOS-2 induction. Exposure of optic nerve astrocytes to interferon gamma and interleukin-1β in culture stimulates NOS-2 production within 24 h.[102] TNF-A appears to be another more relevant cytokine and exposure of astrocytes to TNF-A in culture causes induction of NOS-2.[47] This cytokine along with the TNF receptor-1 has been detected in glaucomatous optic nerve heads. Thus the exposure to cytokines transforms the human optic nerve astrocytes into reactive astrocytes, which contain NOS-2 and have the capability to produce neurotoxic quantities of NO. Besides the important role of cytokines it has also been observed that the human optic nerve astrocytes when exposed to elevated hydrostatic pressure in culture, express elevated NOS-2 levels indicating a direct effect of elevated pressure for induction of NOS-2 in astrocytes.[106] The direct neurotoxic effects of NO on RGC in optic nerve head were further evidenced by the neuroprotective effects of aminoguanidine, a specific NOS-2 inhibitor in rats with chronically elevated IOP.[108]

Thus a large body of evidence suggests that excessive quantities of NO produced by astrocytes and microglia in optic nerve head play a crucial role in the development of optic neuropathy associated with glaucoma. The excess of NO thus produced enters freely into the cells after diffusion through the local microenvironment.[109] It is a free radical of moderate reactivity and after entering the cell leads to the production of highly reactive free radicals such as peroxynitrite after combining with superoxide (a product of mitochondrial metabolism). These highly reactive free radicals are capable of causing massive destruction of cell components and macromolecules.[110]

### Oxidative stress and glaucoma

The ocular tissue is provided with a very efficient antioxidant defense mechanism, which includes reduced glutathione (GSH) and superoxide dismutase-catalase system. Ascorbic acid also has an important protective role and its high concentration has been detected in the vitreous humor,[111] cornea,[112] lacrimal film,[113] central corneal epithelium[114] and aqueous humor.[115] The excessive formation of free radicals and oxidative stress is recognized as an etiopathogenetic factor in many ocular diseases such as cataract,[116] age-related macular degeneration[117] and more recently glaucoma.[118] The glaucoma-affected patients have shown significantly depleted antioxidant potential in the aqueous humor,[119] an increase in serum antibodies against glutathione-*S*-transferase,[120] a decrease in plasma glutathione levels[121] and an increase in lipid peroxidation products in the plasma.[122]

Vascular dysregulation by causing ischemia and reperfusion may be the fundamental pathogenic step in inducing oxidative stress.[123] Under oxidative stress the endothelial functions are altered, especially the production of endothelian-1 and NO.[124,125] The endothelin-1 has been identified as the possible effector in POAG as it brings about changes in the cells of trabecular meshwork by causing vasoconstriction and thereby altering the IOP.[126] Significantly raised endothelin-1 levels have been detected in the aqueous humor of glaucoma patients as compared to normal controls.[57] NO, besides causing increased release of glutamate and neuronal toxicity also reacts with superoxide anion to form the peroxynitrite radical, which adds to the oxidative stress-induced damage.[127] Oxidative stress as a result of free radical accumulation either from aerobic metabolism or vascular dysregulation is known to damage the DNA of the trabecular meshwork cells.[128] As a result, altered adhesion of trabecular cells with the ECM proteins leads to cytoskeleton rearrangements and increased resistance to outflow leading to elevated IOP.[129] Human studies have revealed alterations in aqueous humor drainage following exposure to hydrogen peroxide.[130] More extensive alterations in trabecular cells have been detected in the layers of trabecular meshwork closer to the anterior chamber thus indicating that the exposure to toxic substances such as free radicals, in the anterior chamber plays a crucial role as a pathogenetic factor.

The results of some of the animal experiments have suggested possible benefits of antioxidants in glaucomatous optic neuropathy. In a rat model of glaucoma topical administration of a novel free radical scavenger with esterified ion chelator side groups on a methoxypolyethylene glycol backbone was found to lower IOP by 29.6%. In the same study the RGC loss in rats in response to intravitreal NMDA was also found to be reduced when NMDA was co-administered with the same novel free radical scavanger.[131] BDNF in combination with a nonspecific free radical scavenger was shown to rescue RGC from death in rat eyes with elevated IOP.[132] The results of these studies indicate an association of oxidative stress and development of glaucoma and possible benefits of antioxidants in glaucomatous optic neuropathy.

## Current therapeutic approaches

Glaucomatous optic neuropathy is a chronic process, which progresses over many years. Until recently, modulation of elevated IOP was the only mode of therapeutic intervention. As the glaucomatous changes continue to progress despite well-controlled IOP, development of pressure-independent and preferentially neuroprotective treatment strategies is extremely important. As the understanding of pathophysiological mechanisms involved in glaucomatous optic neuropathy has advanced tremendously, an enormous amount of research has been stimulated for the development of effective neuroprotective strategies.

As a result a variety of therapeutic options have shown efficacy as neuroprotective agents in experimental studies. Besides ocular hypotensive agents the ocular blood flow enhancers such as calcium channel blockers were suggested to provide neuroprotection by improving the optic nerve head blood flow. But there were concerns as these agents reduce the systemic blood pressure and might worsen the optic nerve head ischemia by reducing the perfusion pressure. Carbonic anhydrase inhibitors are also suggested to improve the optic nerve head blood flow.

A variety of agents with antiapoptotic activity have been evaluated for neuroprotective effects in experimental animal models. Both the reversible and irreversible caspase inhibitors were found to protect RGCs in axotomised rats. Erythropoietin, which promotes proliferation and differentiation of bone marrow precursor cells by inhibiting apoptosis, when given by intravitreal injection in an episcleral vessel cautery-induced rat model of glaucoma was found to increase RGC viability.[133] However, the use of these agents, which act by preventing the apoptosis, is actually the treatment of the result rather than the degenerative process itself.

Therapeutic interventions to alter the process of RGC degeneration have also been studied extensively. A variety of neurotrophic factors (BDNF, nerve growth factor), an antioxidant (N-ace-tyl-L-cysteine), and a NOS inhibitor (L-NAME, aminoguinidine) have shown promising neuroprotective effects by modulating the process of RGC degeneration in experimental animals. The NMDA antagonists especially seem to hold promise in glaucoma neuroprotection. NMDA receptor antagonists have largely failed the clinical trials as they act by virtually blocking all the NMDA receptors, and physiological NMDA activity is essential for normal neuronal functions. Memantine, an adamantine derivative, has shown encouraging results as it selectively blocks the excessive receptor activation without affecting the normal receptor activity. Memantine is a noncompetitive, low-affinity, open channel blocker and blocks the receptor-associated ion channel when it is excessively open. As its off-rate is very high it does not accumulate substantially within the channel to interfere with the normal neuronal functions. Memantine is thus well tolerated and has been approved for use in Alzheimer's disease.[134] The results of the efficacy of oral memantine treatment in a Phase 3, randomized, multicenter, placebo-controlled, double-blind clinical trial involving POAG patients are awaited.

In spite of a large number of drugs showing efficacy in animal experiments only one has progressed to the stage of clinical trial. Clearly, the concept of neuroprotective agents playing a major role in glaucoma management is still in infancy. However, a better understanding of the pathophysiological mechanisms involved in glaucoma will undoubtedly lead us to new, safe and effective glaucoma therapy.

## Summary and Conclusions

**Figure 4:**
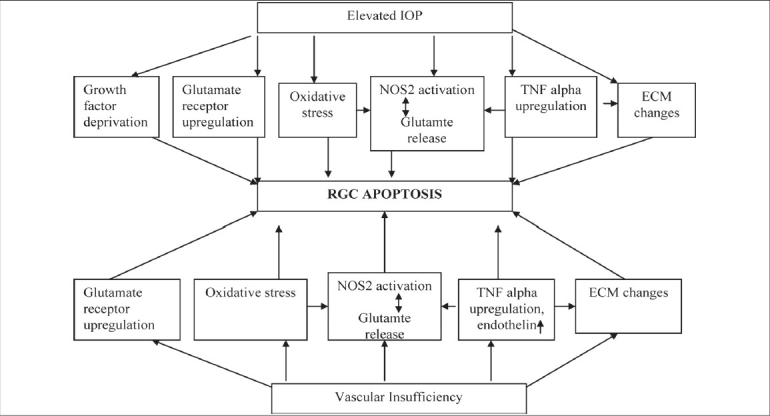
The complex interplay of primary and secondary factors leading to RGC apoptosis in glaucoma. TNF-A - Tumor necrosis factor-alpha, ECM - Extracellular matrix, NOS-2 - Nitric oxide synthase-2

To summarize, the primary factors responsible for apoptotic cell death in glaucoma include not only elevated IOP but also vascular dysregulation, especially in people with NTG. The molecular mechanisms involved largely include glutamate excitotoxicity, increased MMP expression, TNF-alpha upregulation, increased NOS-2 expression and oxidative stress. The complex interrelationship between primary and secondary mechanisms involved in the pathophysiology of glaucoma is shown in Fig. 4. Although current therapeutic approaches, which primarily aim to lower the elevated IOP have shown great efficacy in saving the vision in glaucomatous optic neuropathy, further research to identify and develop pharmacological means with predominant neuroprotective effects is expected to provide better therapeutic options. Limited success so far in the development of effective neuroprotective therapy probably indicates the need of future research that incorporates multiple factors involved in the pathophysiology of neuronal damage in glaucoma.
